# Acute cortical deafness in a child with MELAS syndrome

**DOI:** 10.1007/s10545-016-9929-x

**Published:** 2016-04-07

**Authors:** Marie P. Pittet, Roni B. Idan, Ilse Kern, Nils Guinand, Hélène Cao Van, Seema Toso, Joël Fluss

**Affiliations:** 1 Pediatric Neurology Unit, Pediatric Subspecialties Service, Children’s Hospital, Geneva University Hospitals, Geneva, Switzerland; 2 Child and Adolescent Medicine Department, Children’s Hospital, Geneva University Hospitals, Geneva, Switzerland; 3 Pediatric Nephrology and Metabolism Unit, Pediatric Subspecialties Service, Children’s Hospital, Geneva University Hospitals, Geneva, Switzerland; 4 Division of Otorhinolaryngology, Geneva University Hospitals, Geneva, Switzerland; 5 Pediatric Radiology Unit, Children’s Hospital, Geneva University Hospitals, Geneva, Switzerland

## Abstract

Auditory impairment in mitochondrial disorders are usually due to peripheral sensorineural dysfunction. Central deafness is only rarely reported. We report here an 11-year-old boy with MELAS syndrome who presented with subacute deafness after waking up from sleep. Peripheral hearing loss was rapidly excluded. A brain MRI documented bilateral stroke-like lesions predominantly affecting the superior temporal lobe, including the primary auditory cortex, confirming the central nature of deafness. Slow recovery was observed in the following weeks. This case serves to illustrate the numerous challenges caused by MELAS and the unusual occurrence of acute cortical deafness, that to our knowledge has not be described so far in a child in this setting.

We report an unusual neurological complication of MELAS syndrome in an 11-year-old boy with a genetically confirmed MELAS syndrome (m.3243A > G mitochondrial DNA mutation) for 2 years (Nissenkorn et al. 2000). His basic symptoms were microcephaly, small height, learning disabilities, refractory focal epilepsy, mild weakness and extreme fatigue. After an unusual nocturnal flare-up of seizures, the child appeared, upon awakening, not to respond to any auditory stimuli, and was brought to the emergency room with a presumed subacute profound deafness. On examination, the patient was alert, although he did not show any reactivity to loud sounds. He merely produced meaningless sequences of words such as “il est où papa?” Furthermore, the patient seemed unaware of his hearing disturbances. No other neurological deficits were observed. A conductive hearing loss was ruled out. Auditory evoked potentials from the brainstem were present. A central cause of hearing loss was therefore suspected and a brain MRI performed. This showed multiple and bilateral stroke-like lesions affecting predominantly the superior temporal lobe including both primary auditory cortices, confirming the central nature of the subacute deafness (Fig. 1). High-dose iv L-Arginine was given in addition to his standard treatment of oral Citrulline. Despite progressive hearing recovery within 2 weeks, the patient did not recover fully and exhibits on follow-up, residual language and cognitive deficits. Although hearing impairment in mitochondrial disorders is usually related to cochlear dysfunction, this case emphasizes the possibility of an acute cortical deafness in MELAS due to its unique propensity to simultaneously affect both temporal lobes with stroke-like lesions (Miceli et al. 2008).

**Fig. 1:**
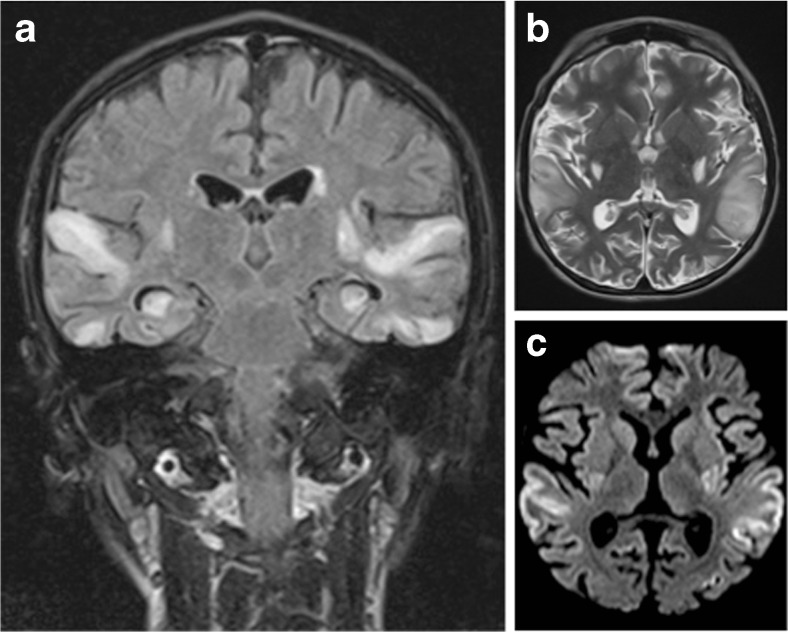
Brain MRI scan 72 h after onset of symptoms. **a** Coronal FLAIR image shows symmetrical high signal intensities lesions in multiple arterial territories. **b** Axial T2-weighted image demonstrating high signal cortical and subcortical lesions bilaterally in the edematous superior temporal gyri. **c** Axial diffusion-weighted image (DWI) demonstrating high signal areas in the same regions
